# CITED2 affects leukemic cell survival by interfering with p53 activation

**DOI:** 10.1038/cddis.2017.548

**Published:** 2017-10-26

**Authors:** Katharina Mattes, Gerbrig Berger, Marjan Geugien, Edo Vellenga, Hein Schepers

**Affiliations:** 1 Department of Hematology, Cancer Research Center Groningen, University Medical Center Groningen, University of Groningen, Groningen, The Netherlands

## Abstract

CITED2 (CBP/p300-interacting-transactivator-with-an-ED-rich-tail 2) is a regulator of the acetyltransferase CBP/p300 and elevated CITED2 levels are shown in a number of acute myeloid leukemia (AML). To study the *in vivo* role of CITED2 in AML maintenance, AML cells were transduced with a lentiviral construct for RNAi-mediated knockdown of CITED2. Mice transplanted with CITED2-knockdown AML cells (*n*=4) had a significantly longer survival compared to mice transplanted with control AML cells (*P*<0.02). *In vitro*, the reduction of CITED2 resulted in increased p53-mediated apoptosis and *CDKN1A* expression, whereas *BCL2* levels were reduced. The activation of p53 upon CITED2 knockdown is not a direct consequence of increased CBP/p300-activity towards p53, since no increased formation of CBP/p300/p53 complexes was demonstrated and inhibition of CBP/p300-activity could not rescue the phenotype of CITED2-deficient cells. Instead, loss of CITED2 had an inhibitory effect on the AKT-signaling pathway, which was indicated by decreased levels of phosphorylated AKT and altered expression of the AKT-pathway regulators *PHLDA3* and *SOX4*. Notably, simultaneous upregulation of *BCL2* or downregulation of the p53-target gene *PHLDA3* rescued the apoptotic phenotype in CITED2-knockdown cells. Furthermore, knockdown of CITED2 led to a decreased interaction of p53 with its inhibitor MDM2, which results in increased amounts of total p53 protein. In summary, our data indicate that CITED2 functions in pathways regulating p53 activity and therefore represents an interesting target for AML therapy, since *de novo* AML cases are characterized by an inactivation of the p53 pathway or deregulation of apoptosis-related genes.

Acute myeloid leukemia (AML) is a genetically heterogeneous disease that is characterized by an accumulation of immature myeloblasts in the bone marrow. Despite the variety in the mutational background, the transcriptional regulator CITED2 (CBP/p300-interacting-transactivator-with-an-ED-rich-tail 2) is found to be upregulated in the majority of AML cases.^1^ As demonstrated by conditional knockout studies, CITED2 is essential for the maintenance of adult hematopoietic stem cells during normal haematopoiesis, whereas it is dispensable in more committed cells.^2^ Notably, recent data strengthened the hypothesis that CITED2 has also critical functions in maintaining human leukemic cells, since knockdown of CITED2 in AML cells inhibited AML engraftment *in vivo*, when competed with control cells.^1^ These findings suggest that CITED2 might have an important role in the survival of the leukemic stem cell (LSCs) – those AML cells that fail to be targeted sufficiently and most likely cause disease relapse.

CITED2 was originally identified and characterized as a protein binding to CH1 domain of the acetyltransferase CBP/p300.^3, 4^ Acetylation of histones and non-histone proteins is crucial for eukaryotic cells to regulate transcription. By binding to CBP/p300, CITED2 was shown to regulate the CBP/p300-mediated transactivation of multiple transcription factors such as HIF1*α*,^3^ NF-*κ*B,^5^ LHX2,^6^ TFAP2,^7^ MYC,^8^ PPAR*α*^9^ and SMAD2.^10^ Presence of CITED2 can enhance or repress the target gene expression of these transcription factors. In case of HIF1*α* and NF-*κ*B, CITED2 has been described to compete for p300 and thereby block HIF1*α*- and NF-*κ*B-mediated transcription.^3, 5^ For most other transcription factors, CITED2 acts as a co-activator of transcription by recruiting CBP/p300 to the transcription factor target sites. Whereas increased expression of CITED2 has been described to facilitate MYC- and PPAR*α*-mediated cell-cycle progression,^8^ low expression of CITED2 is associated with an increase of cell death, for example by impaired SMAD2- and TFAP2 signaling.^10^ Interestingly, loss of CITED2 expression has been reported to lead to increased activation of the tumor suppressor protein p53,^12^ which is acetylated and co-activated by CBP/p300. Wu *et al.*^12^ suggested increased formation of p53-CBP/p300 complexes in the absence of CITED2. However, other studies reported decreased p300 function in CITED2-deficient cells,^13^ and therefore the role of CITED2 in p53 signaling is still unclear.

Here we report that loss of CITED2 severely impairs AML cell survival by induction of p53-mediated apoptosis. Interestingly, loss of CITED2 activates the p53 pathway independent of CBP/p300-mediated acetylation of p53. Instead, reduction of CITED2 levels impairs cell survival by interfering with the AKT signal transduction pathway.

## Results

### Knockdown of CITED2 in CD34^+^ AML cells impairs leukemia development

**Figure 1:**
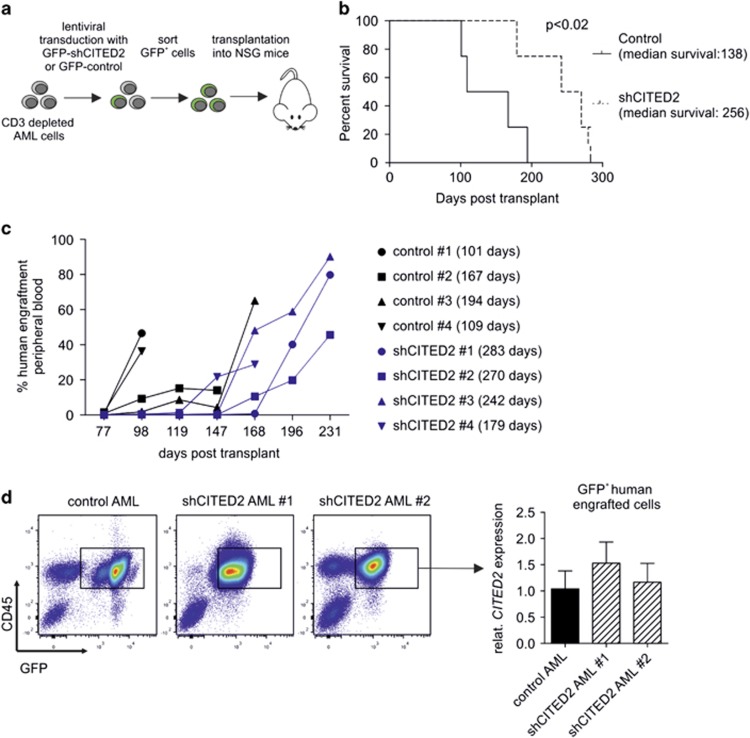
Knockdown of CITED2 in AML cells impairs leukemia development. (**a**) Schematic overview of lentiviral shRNA transductions for mice transplantation experiments. (**b**) Kaplan–Meier curve indicating the survival of mice transplanted with sorted control- or shCITED2- transduced CD3-depleted AML cells. *n*=4, *P*<0.02. (**c**) NSG mice were analyzed for the presence of human engraftment at indicated time points after transplantation with control- or shCITED2-transduced AML cells. Percentage of human CD45^+^ cells in peripheral blood is shown. Survival of the individual mice is indicated in brackets. (**d**) FACS plots indicating sorting strategy for human GFP^+^ cells from sacrificed NSG mice (left panel) and Q-PCR analysis for *CITED2* in sorted cells (right panel) are shown. Error bars indicate s.d. of Q-PCR triplicates

In order to demonstrate that CITED2 truly impacts the survival and maintenance of leukemia initiating cells (LICs, or LSCs), AML cells were transduced with a lentiviral GFP-vector expressing a short hairpin targeting CITED2 (shCITED2) or a control vector. The hairpin was selected based on previous data demonstrating that various shCITED2-constructs resulted in similar phenotypes.^1^ After transduction, GFP-positive cells were sorted and subsequently transplanted into NSG mice (*n*=4) (Figure 1a). Mice which only received sorted shCITED2-transduced leukemic cells have a significantly longer survival as compared to mice that only received control-transduced leukemic cells (138 *versus* 256 days *P*<0.02, Figure 1b). Eventually, engraftment of human AML cells and leukemia development was observed in both control- and shCITED2 mice (Supplementary Figure S1); however, the progression in shCITED2 mice was delayed (Figure 1c). It appeared that the GFP-positive shCITED2-AML cells that eventually did engraft did not show a reduction of CITED2 levels and therefore most likely have escaped sufficient knockdown of CITED2 (Figure 1d), indicating that CITED2 knockdown impacts the survival and maintenance of leukemia initiating cells.

### Loss of CITED2 triggers apoptosis in leukemic cells

**Figure 2:**
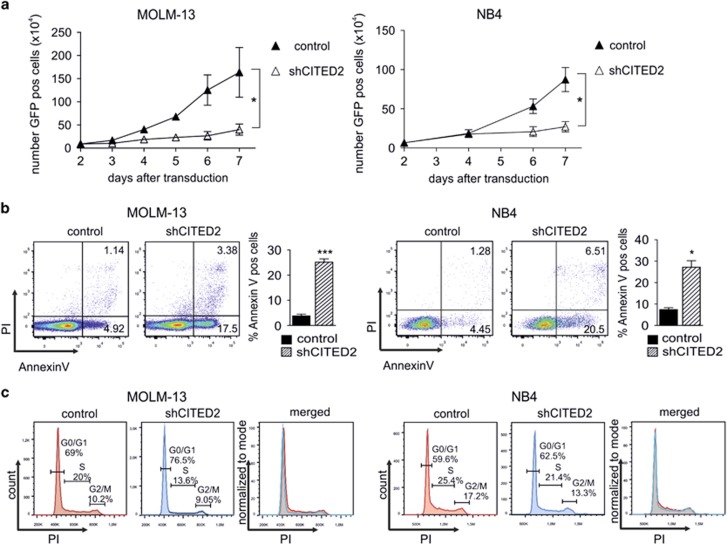
Loss of CITED2 triggers apoptosis in leukemic cells. (**a**) The leukemic cell lines MOLM-13 and NB4 were transduced with a GFP-expressing control- or shCITED2 construct. The number of GFP^+^ cells over time is shown. Error bars indicate s.d. of three individual experiments; *P<0.05. (**b**) Annexin V staining of NB4 and MOLM-13 cells 4–5 days after transduction with shCITED2. FACS plots of an individual experiment (left panels) and average percentage of Annexin V-positive cells from multiple experiments (right panels) is shown. Error bars indicate s.d. (*n*=6 MOLM-13, *n*=4 NB4). ***P<0.001, *P<0.05. (**c**) Cell-cycle analysis of control- and shCITED2-transduced MOLM-13 (left panel) and NB4 cells (right panel)

Similar suppressive effects of CITED2 reduction were observed in the leukemic cell lines NB4 and MOLM-13 (Figure 2a, Supplementary Figure S2A). To gain a first insight into the cause of reduced cell expansion, the effects of CITED2 knockdown on apoptosis and cell-cycle distribution were analyzed in NB4 or MOLM-13 cells. A strong increase of Annexin V^+^ cells was observed in shCITED2 cells compared to control cells (Figure 2b, Supplementary Figure S2B), whereas no significant changes in cell-cycle distribution could be demonstrated (Figure 2c, Supplementary Figure S2C).

### shCITED2-mediated cell death is triggered by a p53-dependent pathway

**Figure 3:**
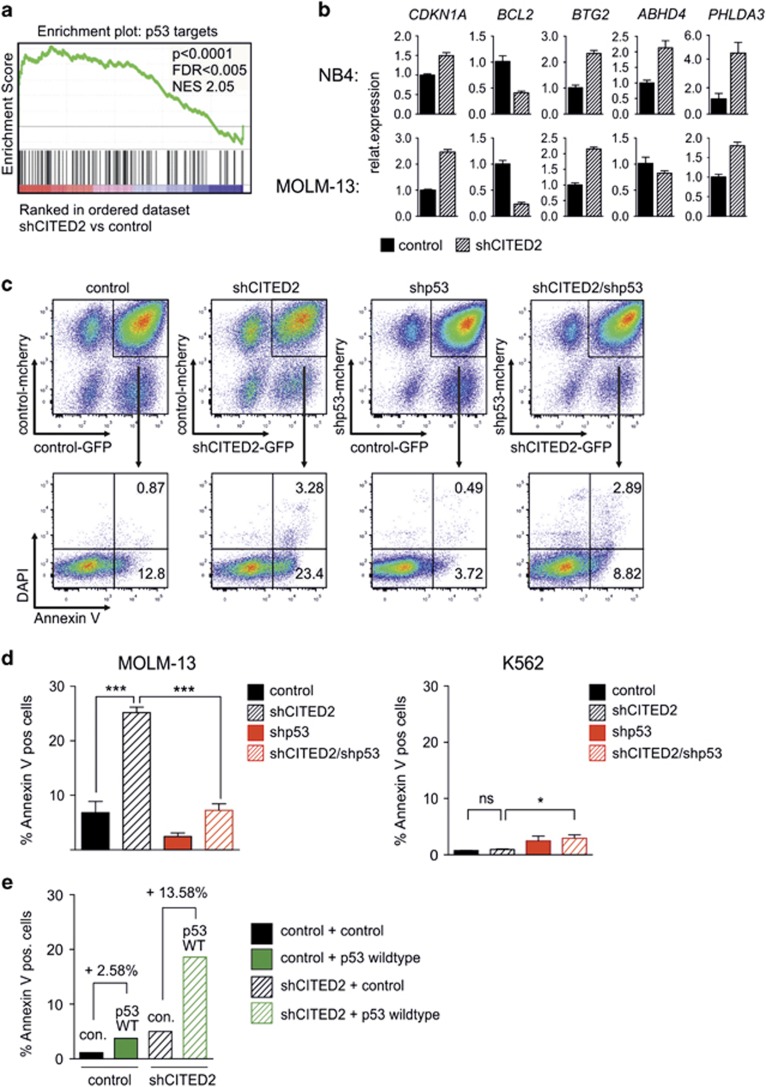
shCITED2-mediated cell death is triggered by a p53-dependent pathway. (**a**) Gene set enrichment analysis (GSEA) of genome-wide gene expression from CD34^+^ cord blood cells transduced with a control- or shCITED2 vector revealed an enrichment of p53 target genes. (**b**) Q-PCR of p53 targets in NB4 and MOLM-13 cells 4–5 days after transduction with the shCITED2 vector. Error bars indicate s.d. of triplicates from representative Q-PCR. (**c**) MOLM-13 cells were double-transduced with GFP and mCherry expressing shRNA vectors to knockdown CITED2 and/or p53 and stained for Annexin V 4–5 days after transduction. Representative FACS plots indicating the percentage of Annexin V-positive cells are shown. (**d**) Percentage of Annexin V-positive cells in shCITED2/shp53-transduced MOLM-13 (*n*=5) and K562 (*n*=3) cells 5 days after transduction is shown. Error bars indicate s.d. *P<0.05, ***P<0.001; n.s.= not significant. (**e**) MOLM-13 cells were co-transduced with a construct for *CITED2* knockdown and *p53* overexpression. Percentage of Annexin V-positive cells 3 days after transduction is indicated

Previously, microarray analysis of shCITED2-transduced *versus* control-transduced CD34^+^ cord blood cells was performed to identify the molecular pathways that are particularly dependent on CITED2 levels (GSE47218).^1^ Further analysis of this data set revealed a significant enrichment of p53 target genes among the upregulated genes after CITED2 knockdown (Figure 3a). Gene expression analyses by Q-PCR in shCITED2-transduced NB4 and MOLM-13 cells confirmed an upregulation of several p53 targets such as *CDKN1A*, *BTG2*, *ABHD4* and *PHLDA3*, whereas the p53-regulated anti-apoptotic protein *BCL2* was strongly down regulated (Figure 3b,Supplementary Figure S3A). Simultaneous knockdown of p53 and CITED2 rescued the increased apoptosis levels in NB4 and MOLM-13 cells (Figures 3c and d, Supplementary Figure S3B), while lentiviral overexpression of p53 potentiated shCITED2-mediated apoptosis (Figure 3e). Importantly, decreasing CITED2 levels had no impact on apoptosis levels of leukemic cell lines that are devoid of p53, such as K562 (Figure 3d, right panel).

### Activation of p53 signaling in CITED2 knockdown cells is not due to direct CBP/p300-mediated p53 acetylation

**Figure 4:**
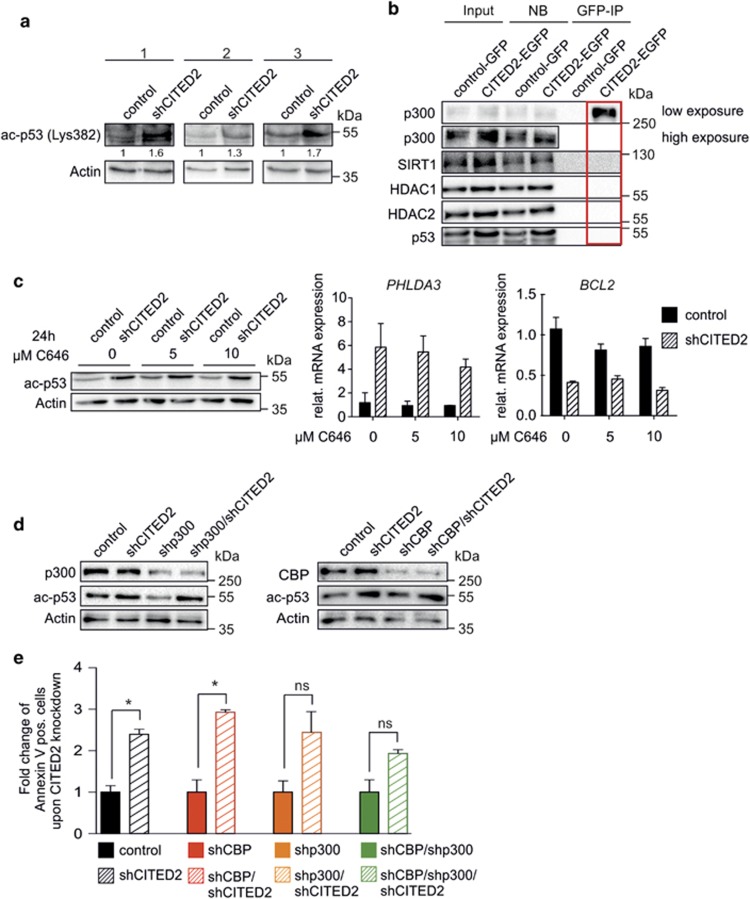
Increased p53 activation in CITED2-knockdown cells is not directly mediated by CBP/p300. (**a**) Western blot analysis for ac-p53 (Lys382) of sorted GFP^+^ NB4 cells transduced with a control- or shCITED2-expressing construct. Cells were sorted 4 days after transduction. Numbers indicate quantification of the ac-p53 signal normalized to Actin. (**b**) GFP-coimmunoprecipitation (IP) experiment of CITED2:EGFP-expressing NB4 cells and western blot analysis for indicated antibodies. Red rectangle highlights the lane showing interactions between CITED2 and the indicated proteins. (**c**) NB4 cells were transduced and sorted as in (**a**). Cells were treated with indicated concentration of the CBP/p300 inhibitor C646 for 24 h before sorting. Western blot analysis for ac-p53 (left panel) and Q-PCR analysis for *PHLDA3* (middle panel) and *BCL2* (right panel) expression is shown. Error bars indicate s.d. of Q-PCR triplicates. (**d**) NB4 cells were simultaneously transduced with indicated combinations of shRNA constructs for downregulation of *CITED2*, *EP300* and *CBP*. Western blot analysis for indicated antibodies of sorted cells is shown. (**e**) Annexin V staining of MOLM-13 cells transduced as in (**d**). Percentage of Annexin V-positive cells is indicated. Error bars indicate s.d. of two individual experiments; *P<0.05

As activated p53 signaling turned out to be a crucial factor in the shCITED2-mediated cell death and CITED2 has been described to co-regulate binding of CBP/p300 to its targets, we questioned whether CBP/p300-mediated acetylation on C-terminal lysine 382 of p53^14^ is involved. In shCITED2-transduced NB4 cells, increased acetylation of p53 on the C-terminal lysine 382 was observed compared to control cells (Figure 4a). Next, it was investigated whether CITED2 directly interferes with p53 acetylation by either (i) recruiting deacetylases (HDACs, SIRTs) to the p300/p53 complex, or (ii) by blocking the binding of CBP/p300 to p53. By performing co-immunoprecipitation (IP) assays with lysates from transduced NB4 cells, binding of CBP/p300 to CITED2 as well as binding of CBP/p300 to p53 was observed (Supplementary Figure S4A), but no evidence was obtained for a CBP/p300/CITED2/p53 interaction (Figure 4b). In addition, no interaction of CITED2 with HDAC1, HDAC2, or the acetyltransferase P/CAF could be demonstrated (Figure 4b, Supplementary Figure S4B). The knockdown of CITED2 did not lead to an increased binding of p53 to CBP or p300 (Supplementary Figure S4C). Finally it was shown that treatment of leukemic cells with the CBP/p300 inhibitor C646 for 24 h did not rescue the shCITED2-mediated effects on p53 acetylation, or *PHLDA3*- and *BCL2* expression (Figure 4c). These results suggest that the effects of shCITED2 are independent of direct CBP/p300-mediated p53 activation. This was confirmed by simultaneous transduction of shCITED2 cells with short hairpins against p300, CBP or both, which did not affect levels of p53 acetylation (Figure 4d). Furthermore, knockdown of CITED2 in CBP/p300-deficient cells still led to an approximately twofold increase in apoptosis (Figure 4e). These data propose that p53 activation in shCITED2 cells is not a direct consequence of interfering with its acetylation state. This hypothesis was further supported by the fact that interference with other acetyltransferases that target p53 (P/CAF,^15^ GCN5,^16^ TRRAP,^17^ TIP60^18, 19^ or MOZ^20^) did not rescue the phenotype of CITED2-low cells. There was no increase in GCN5 binding to ac-p53 in NB4 cells with CITED2 knockdown (Supplementary Figure S4D) and treatment of shCITED2-transduced MOLM-13 cells with the TIP60/MOZ inhibitor MG-149 could not rescue the apoptotic phenotype of cells upon CITED2 knockdown (Supplementary Figure S4E).

### CITED2 knockdown mediates a p53/PHLDA3/BCL2-dependent decrease in AKT signaling

**Figure 5:**
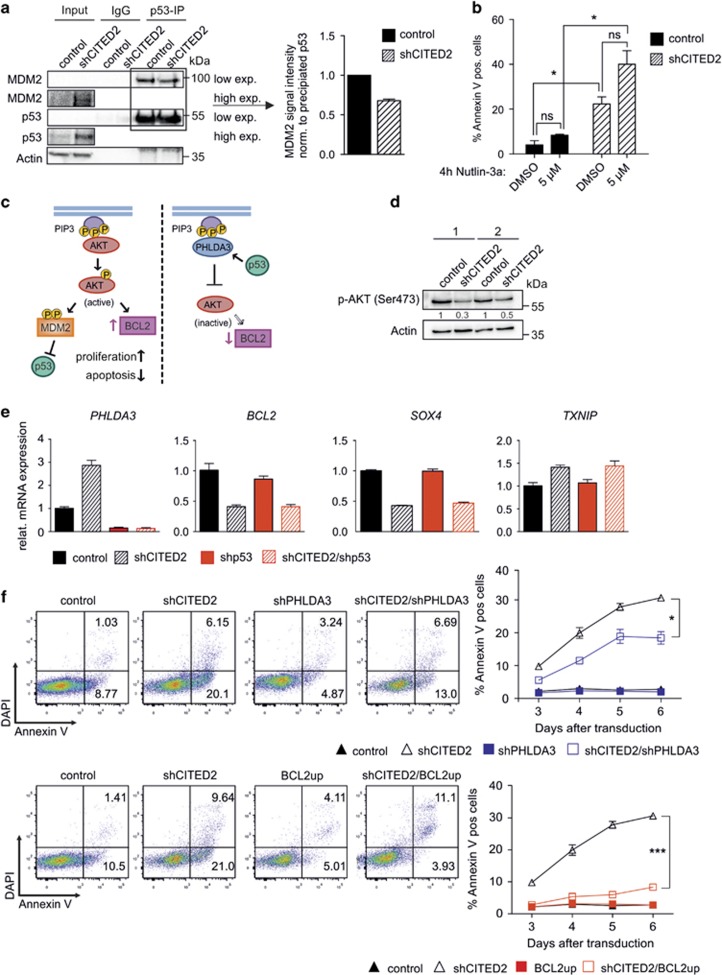
CITED2 knockdown mediates decrease in AKT-signaling in a p53/PHLDA3/BCL2-dependent manner (**a**) p53 co-IP of sorted control- or shCITED2-transduced MOLM-13 cells with subsequent western blot analysis for MDM2 (left panel). Right panel indicates quantified MDM2–p53 interaction. Error bars indicate s.d. from two individual experiments. (**b**) Percentage of Annexin V-positive cells of control- or shCITED2-transduced MOLM-13 cells after incubation with indicated concentration of the inhibitor Nutlin3a for 4 h. *n*=2; error bars indicate s.d.; *P<0.05. (**c**) Schematic excerpt of the AKT signaling pathway: AKT activation requires binding of AKT to second messenger molecules (PIP3) at the cell membrane which eventually leads to expression of the pro-survival gene *BCL2* and increased formation of MDM2–p53 complexes leading to p53 degradation. Binding of the p53 target PHLDA3 to PIP3 interferes with that process. (**d**) Western blot analysis of NB4 cells for p-AKT expression after lentiviral knockdown of CITED2. (**e**) Q-PCR for indicated genes in shCITED2-, shp53 or shCITED2/shp53-transduced cells. Error bars indicate Q-PCR triplicates. (**f**) MOLM-13 cells transduced with a short hairpin construct against *CITED2* in combination with a short hairpin against *PHLDA3* or a lentiviral construct for overexpression of *BCL2* were stained for Annexin V. FACS plots of an individual experiment (left panels) and average percentage of Annexin V-positive cells at several days after transduction (right panels) are shown. Error bars indicated s.d. (*n*=2); *P<0.05, ***P<0.001

As depicted in Figure 5a, knockdown of CITED2 also leads to an increased amount of total p53 protein. The ubiquitin ligase MDM2 has a central role in regulating p53 stability by binding to p53 and thereby either directly inhibiting its transcriptional activity or promoting proteasome-mediated degradation of p53 (reviewed in Kruse and Gu^21^ and Manfredi^22^). By performing IP in MOLM13 cells, it was demonstrated that knockdown of CITED2 results in decreased interaction between MDM2 and p53 (Figure 5a). In line with these data, shCITED2-transduced cells had an increased sensitivity to treatment with Nutlin3a, an inhibitor of the MDM2–p53 interaction (Figure 5b). The pro-survival AKT signaling pathway^23, 24^ is promoting p53-inhibiting functions of MDM2 (Figure 5c). Interestingly, knockdown of CITED2 resulted in strong increase in mRNA expression of *PHLDA3* (Figure 3b), a known repressor of AKT signaling.^25^ PHLDA3 interferes with binding of AKT to second messenger molecules at the cell membrane and thus prevents AKT phosphorylation and activation (Figure 5c). In line with these data, decreased AKT phosphorylation, and thus decreased AKT activation was observed in CITED2-knockdown cells by western blotting (Figure 5d). Besides upregulation of *PHLDA3*, upregulation of the AKT-inhibitor *TXNIP*^26^ and downregulation of the AKT-activator *SOX4*^27^ was observed (Figure 5e). Interestingly, simultaneous knockdown of p53 rescued the shCITED2-mediated increase in *PHLDA3* expression, whereas shCITED2-mediated alteration of *BCL2*, *SOX4* and *TXNIP* was found to be p53-independent (Figure 5e). To further investigate the involvement of the AKT-signaling pathway in the shCITED2-mediated phenotype, transduced shCITED2 cells were simultaneously transduced with a short hairpin against *PHLDA3* or a lentiviral construct overexpressing *BCL2* (Supplementary Figure S5). Simultaneous knockdown of *CITED2* and *PHLDA3* partially rescued the shCITED2-mediated induction of cell apoptosis (Figure 5f upper panel), indicating that the increased expression of *PHLDA3* contributed to the CITED2-mediated effects on cell survival. Strikingly, simultaneous upregulation of *BCL2* in CITED2 knockdown cell could almost completely rescue the apoptotic phenotype (Figure 5f lower panel). These data demonstrate that both upregulation of *PHLDA3* and downregulation of *BCL2* are critical events in shCITED2-mediated apoptosis in AML cells.

## Discussion

The results of our study demonstrate that reduction of CITED2 levels impairs the survival of leukemic cells, in part due to induction of p53-mediated apoptosis. Notably, AML cells are characterized by elevated *CITED2-*expression and the present study highlights the importance of CITED2 levels in regulating cell survival pathways. It appeared that the activation of the p53 pathway is not due to increased CBP/p300-mediated p53 acetylation, which can stabilize p53 protein expression. Although an overall increase of acetylated p53 in CITED2-deficient cells was detected, treatment of cells with the CBP/p300 inhibitor C646 as well as RNAi-mediated knockdown of *CBP/P300* could not rescue the effect on p53-mediated apoptosis and target gene expression, indicating that p53 activation upon CITED2 knockdown is not due to increased activity of CBP/p300 towards p53. This is in contrast to a previous study^12^ suggesting an increased formation of CBP/p300/p53 complexes in CITED2-deficient cells. Also increased deacetylation by SIRT1 or HDAC1 and HDAC2 in CITED2-knockdown cells could not be demonstrated, suggesting that the detected increase in acetylated p53 is mainly a result of an increased amount of total p53 protein.

Importantly, reactivation of p53 function represents a relevant strategy for AML therapy, since approximately 90% of *de novo* AML cases do not show a mutation in the p53 gene,^28^ but are rather characterized by an inactivation of the p53 pathway or deregulation of apoptosis-related genes.^29^ We demonstrated that reduction of CITED2 levels results in a decreased interaction between p53 and its inhibitor MDM2. Disruption of the MDM2–p53 interaction has been reported to serve as a promising tool in AML therapy^30, 31^ and various small molecules targeting p53 are currently implemented in the treatment of hematological malignancies to improve the efficiency of conventional cytotoxic drugs.^32^ One of the molecules used for targeting p53 is the MDM2–p53 inhibitor Nutlin3a and in this study we demonstrated that reduction of CITED2 expression sensitizes AML cells to Nutlin3a treatment.

Inactivation of p53 signaling can be caused by aberrant activation of the AKT signaling pathway,^33^ which controls proliferation and differentiation of hematopoietic cells. Notably, constitutive activation of AKT (indicated by increased phosphorylation of AKT on Ser473 and Thr308) is found in 50–70% of AML patients.^34^ Multiple studies in the course of the last years demonstrated that aberrant activation of AKT signaling plays a critical role for the survival and maintenance of AML cells (reviewed in Fransecky *et al.*,^35^ Park *et al.*^36^ and Birkenkamp *et al.*^37^). Whereas mutations of components involved in AKT downstream signaling are rarely found in AML, mutations of membrane-bound proteins upstream of AKT that trigger its activation are common.^28^ A frequent source of AKT deregulation in AML are mutations in receptor tyrosine kinases such as presence of FLT3-ITD^38^ or aberrant IGF1/IGF1-R signaling.^39^ Compounds that interfere with AKT signaling are currently used for AML therapy; however, further understanding of this pathway in order to improve drug development is still needed.

**Figure 6:**
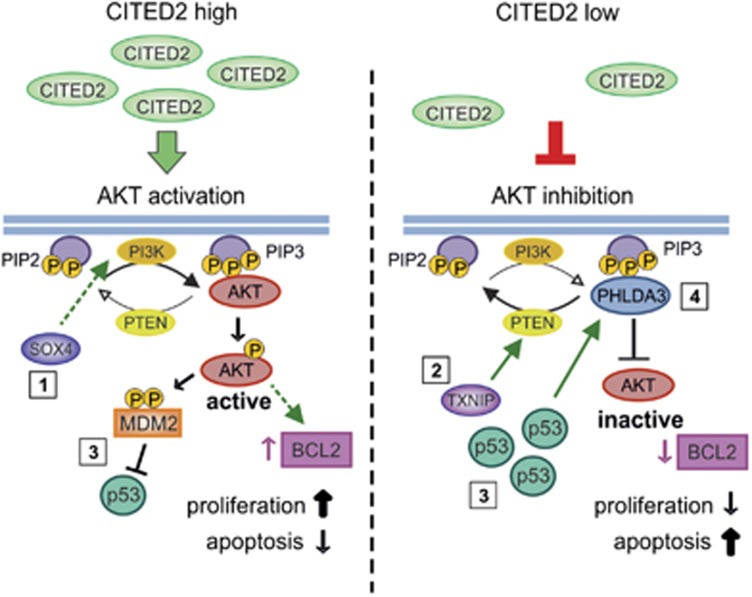
Knockdown of CITED2 interferes with AKT-signaling on multiple levels Reduction of CITED2 levels affects multiple factors involved in regulating AKT-signaling: (1) Knockdown of *CITED2* leads to reduced expression of the transcription factor *SOX4* that stimulates PI3K/AKT signaling. (2) CITED2-low cells show a higher expression of the Thioredoxin-interacting protein (*TXNIP*) that reactivates oxidized PTEN and thereby opposes AKT downstream signaling. (3) Knockdown of *CITED2* results in decreased interaction between p53 and its inhibitor MDM2, leading to increased amounts of p53 protein. (4) Reduction of *CITED2* leads to increased expression of the p53 target gene PHLDA3 that competes with AKT for the binding of membrane lipids. Decreased AKT signaling results in decreased expression of the pro-survival gene *BCL2* and increased apoptosis

Notably, our present study indicates that CITED2 is acting in p53-dependent, as well as in p53-independent pathways. Reduction of CITED2 levels was shown to interfere with AKT signaling on multiple levels (summarized in Figure 6). Whereas upregulation of the p53-target gene and AKT-inhibitor *PHLDA3* appeared to be a direct consequence of increased p53 protein levels, upregulation of the AKT-inhibitor *TXNIP* and downregulation of the AKT-activator *SOX4* upon *CITED2* knockdown occurred in a p53-independent manner. These findings are in line with p53-ChIP-Seq data indicating that there is no p53-binding at the *TXNIP*- and *SOX4* promoter region.^40^ The transcription factor SOX4 binds to the promoter region of multiple components of the AKT signaling pathways and facilitates their transcription,^27^ whereas TXNIP has been shown to play a role in activating PTEN^26^ – an important antagonist of AKT activation – which can directly interfere with MDM2.^41, 42^ Interestingly, *SOX4* and *CITED2* expression are both induced by TGF-*β* signaling, and the CITED2-interaction partner SMAD2 was found to bind to the SOX4 promoter region, indicating that SOX4 and CITED2 might indeed function in the same signaling pathway.^43^ Further evidence for a potential CITED2-SOX4 axis came from the finding that decreased levels of the CITED2 inhibitor PU.1^1^ had a synergistic effect with SOX4 overexpression in promoting murine myeloid leukemia.^44^

Moreover, we identified the p53-independent downregulation of *BCL2* to be a crucial factor in the apoptotic phenotype of CITED2-knockdown cells. BCL2 overexpression is a hall mark of AML and does not only contribute to AML pathogenesis but also plays an important role in therapy resistance (reviewed in Tzifi *et al.*^45^). Previous *in vitro* studies demonstrated that AML cells are very sensitive to BCL2 inhibition.^46^ Notably, AKT signaling is closely interconnected with members of the BCL2 family^47^ and a combination of AKT- and BCL2-inhibitors in AML treatment could represent a beneficial combination.^48^ Therefore, components that regulate these pathways represent potential therapeutic targets.

In summary, we identified that reduction of CITED2 expression interferes with signaling pathways commonly deregulated in AML. Therefore, CITED2 represents a novel, potential target for AML therapy and it will be of interest to study the detailed mechanisms of CITED2-mediated interference with AKT/BCL2/p53 signaling.

## Materials and methods

### Isolation of primary cells

Cord blood (CB) CD34^+^ cells were derived from neonatal cord blood from healthy full-term pregnancies after informed consent from the Obstetrics departments of the Martini Hospital and University Medical Center in Groningen, The Netherlands. AML blasts from peripheral blood cells or bone marrow cells from untreated patients with AML were studied after informed consent and the protocol was approved by the Medical Ethical Committee. CB and AML mononuclear cells were isolated by density gradient centrifugation and CD34^+^ cells were selected using the MACS CD34 microbead kit on autoMACS (Miltenyi Biotec, Amsterdam, the Netherlands).

### Cell cultures

The NB4 and MOLM-13 leukemic cell-lines (DSMZ: ACC 207 and 554) were cultured in RPMI1640 (Lonza, Leusden, the Netherlands, containing L-glutamine) supplemented with 10% FCS and 100 U/ml penicillin/streptomycin. CD34^+^ selected CB cells were cultured in HPGM (Lonza) supplemented with SCF, FLT-3 ligand and N-Plate (Amgen, Breda, the Netherlands) (100 ng/ml each) prior to transductions. AML cells were cultured in HPGM (Lonza) supplemented with 20 ng/ml IL-3, G-CSF and Nplate prior to transductions. 293T cells were cultured in DMEM (Lonza) supplemented with 10% heat-inactivated FCS and 100 U/ml penicillin/streptomycin.

### Lentiviral vectors and transductions

Lentiviral vectors for shRNA expression: shRNA against CITED2 was obtained from Open Biosystem (Cambridge, UK, Clone IDs: TRCN0000015654 and TRCN0000015655) and cloned into a pLKO.1 GFP (kind gift from Prof. Larson, Lund University) and pLKO.1 mCherry vector (obtained by swapping GFP with mCherry). The CITED2 hairpins were chosen based on previous studies demonstrating that these hairpins had similar effects when targeting CITED2.^1^ The P300 PGK-TurboRFP shRNA vector was obtained from GE Healthcare (Cambridge, UK, #V3SH11243-00EG2033). The PHLDA3 oligomers were obtained from Invitrogen (Thermo Fisher Scientific, Landsmeer, the Netherlands) and cloned into the ‘miR-E’ backbone.^49^ The pLKO.1 mCherry shCBP construct was kindly provided by Walderik Zomerman (UMCG, University of Groningen). Short hairpin sequences used in this study are listed in Supplementary table 2. A lentiviral vector expressing BCL2 was constructed by inserting BCL2 cDNA into the lentiviral expression vector pRRL-IRES-blueberry. Lentiviral particles were produced as described before.^50^ After 24 h, medium was changed to HPGM and after 12 h, supernatant containing lentiviral particles was harvested and stored at −80 °C. Cells were transduced with lentiviral particles in the presence of 4 *μ*g/ml polybrene. For AML and cord blood cells, virus particles were concentrated using CentriPrep Ultracel YM-50 Filter Units (Merck Millipore, Billerica, MA, USA) and cells were transduced in two consecutive rounds of 12 h.

### FACS analysis

All FACS analyses were performed on an LSRII (Becton Dickinson, Franklin Lakes, NJ, USA) or BD Accuri (BD Biosciences, Vianen, the Netherlands) flow cytometer and data were analyzed using FlowJo software. Cells were sorted on a MoFLo XDP or Astrios (DakoCytomation, Carpinteria, CA, USA). For Annexin V staining, cells were harvested 4–7 days after lentiviral transduction, washed in calcium buffer (10 mm HEPES/NaOH, pH 7.4, 140 mm NaCl, 2.5 mm CaCl_2_), and stained with 3 *μ*l Annexin V antibody (IQ-products, Groningen, the Netherlands) in 60 *μ*l calcium buffer for 20 min at 4 °C. Cells were subsequently washed and resuspended in calcium buffer for analysis. For cell cycle analysis, cell were resuspended in 200 *μ*l PI-working solution (4 mM trisodium citrate, 20 *μ*g/ml propidium iodide, 1% v/v Triton X-100, 0,1 mg/ml RNaseA), incubated for 30 min dark at 4 °C, and analyzed (Ex/Em: 488/610). For some experiments, cells were treated with following inhibitors as indicated in the figures: C646 (BioVision, Uithoorn, the Netherlands, 1948), Nutlin-3a (Sigma-Aldrich, St. Louis, MO, USA, SML0580), MG-149 (kindly provided by FJ Dekker, University of Groningen).

### Immunoblotting

Equal amounts of sorted cells were boiled in Laemmli sample buffer for 10 min and separated by SDS–PAGE (7.5–10% gels) according to standard protocols. Proteins were transferred onto Immobilon-P PVDF membran (Millipore) by wet blotting overnight. Membranes were blocked with 5% BSA/PBS prior to primary antibody incubation. Signal detection was carried out using HRP-coupled secondary antibodies (DAKO, Amstelveen, the Netherlands) and the SuperSignal West Dura Extended Duration Substrate (Thermo Fisher Scientific). Membranes were scanned on a Bio-Rad ChemiDoc MP Imaging System and signal intensities were quantified using Image Lab software (Bio-Rad, Veenendaal, the Netherlands). For detection of p-AKT, cells were incubated with 100 ng/ml Igf2 for 12 min prior to resuspension in Laemmli buffer. Primary antibodies for immunoblotting were: p53 (DO-1, sc-126 Santa Cruz, Huissen, the Netherlands), acetyl-p53 (Lys382, 2525 S, Cell Signaling, Leiden, the Netherlands), p300 (N15, sc-584, Santa Cruz), CBP (D6C5, 7389, Cell Signaling), PCAF/KAT2B (ab176316 (EPR2670(N)), abcam/RabMab, Uithoorn, the Netherlands), SIRT1, (B10, sc-74504, Santa Cruz), HDAC1 (10E2, 5356, Cell Signaling), HDAC2 (3F3, 5113, Cell Signaling), GFP (B2, SC-9996, Santa Cruz), Pospho-AKT (Ser473, D9E, 4060, Cell Signaling), *β*-Actin (C4, SC-47778, Santa Cruz), MDM2 (sc-965, Santa Cruz), GCN5L2 (C26A10, 3005, Cell Signaling).

### RNA isolation and Q-PCR

Total RNA was isolated using the RNeasy Micro Kit (QIAGEN, Venlo, the Netherlands) following the manufacturer’s instructions. RNA was reverse transcriped using the iScript cDNA synthesis kit (Bio-Rad). Real-Time PCR was performed on a CFX Connect System (Bio-Rad) using the SsoAdvanced SYBR Green Supermix (Bio-Rad). Data were quantified using CFX Manager software (Bio-Rad) and normalized to values of the housekeeping gene RPS11, RPL27 or HPRT. Primer sequences are listed in Supplementary information table 1.

### Co-immunoprecipitation

Equal numbers of sorted cells were resuspended in lysis buffer (25 mM Tris-HCL [pH=7.5], 137 mM NaCl, 1 mM EDTA, 1% NP40, 1 mM DTT, 10% Glycerol) supplemented with 1 × CLAP protease-inhibitor cocktail (Chymostatin, Leupeptin, Antipain and Pepstatin A, 1 *μ*g/ml each), 0.1 mM PMSF and 5 mM sodiumbutyrate. IP targeting endogenous protein: Cell lysates were incubated for 4 h at 4 °C with 2 *μ*g antibody. Immunoprecipitation was carried out using protein G beads (Dynabeads; Invitrogen, Landsmeer, the Netherlands) overnight at 4 °C. IP targeting GFP-fusion proteins: Cell lysates were incubated with 25 *μ*l GFP-trap_MA beads (Chromotek, Planegg-Martinsried, Germany) overnight at 4 °C. Immune complexes were washed three times with lysis buffer, boiled in SDS lysis buffer and further analyzed by immunoblotting as described above. Primary antibodies for Co-IP: acetyl-p53 (Lys382; Cell Signaling Technology; catalog number 2525L).

### *In vivo* transplantations into NSG mice

Eight- to 10-week-old female NSG (NOD.Cg-Prkdcscid Il2rgtm1Wjl/SzJ) mice were purchased from Charles River Laboratory (L'Arbresle Cedex, France) and bred in house. Mouse experiments were performed in accordance with national and institutional guidelines and all experiments were approved by the Institutional Animal Care and Use Committee of the University of Groningen (IACUC-RuG). Before transplantations, mice were sublethally irradiated (2.4 Gy). Following irradiation mice received 3.5 g/l neomycin in their drinking water for 2 weeks. For the transplantation of human AML cells, GFP^+^ CD3^−^ cells were sorted. The mice were injected with 1.5 × 10^5^ sorted GFP^+^ CD3^−^ cells into the tail vein immediately after sorting. Human cell engraftment was analyzed in the peripheral blood (PB) by flow cytometry after 6–40 weeks of transplantation. AML cells were analyzed by exome sequencing and revealed multiple mutations including NPM1 (VAF 0,35), FLT3-ITD (VAF 0,48) and TET2 (VAF 0,45). VAF: variant allele frequency.
