# Supplementary material for: CITED2 affects leukemic cell survival by interfering with p53 activation
Source: Cell Death Dis. 2017 Oct 26;8(10):e3132–. doi: 10.1038/cddis.2017.548 (PMC5680917; doi:10.1038/cddis.2017.548)
Supplement: Supplementary Information [file cddis2017548x1.pdf]

Supplementary Information

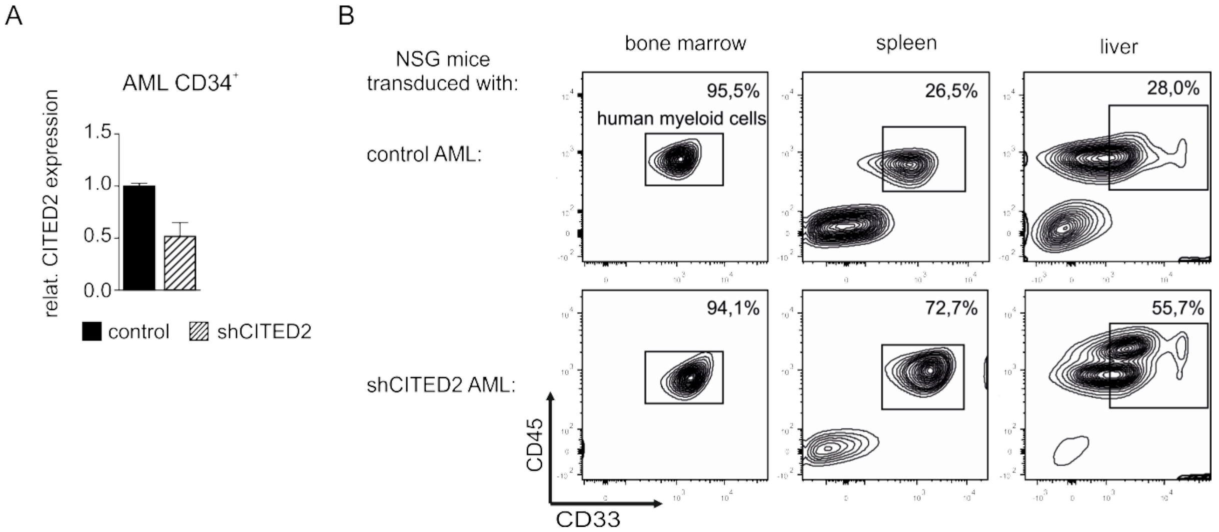

Supplementary Figure S1 (Supplement to Figure 1)

Loss of CITED2 impairs leukemic cells survival

(A) Q-PCR analysis for CITED2 in AML $CD34^+$ cells transduced with a lentiviral construct for downregulation of CITED2. Error bars indicated triplicates of representative Q-PCR. **(B)** Representative FACS plot of cells isolated from NSG mice at day of sacrifice that have been previously transplanted with control- or shCITED2-transduced AML cells. The percentage of engrafted human myeloid cells in indicated organs is depicted.

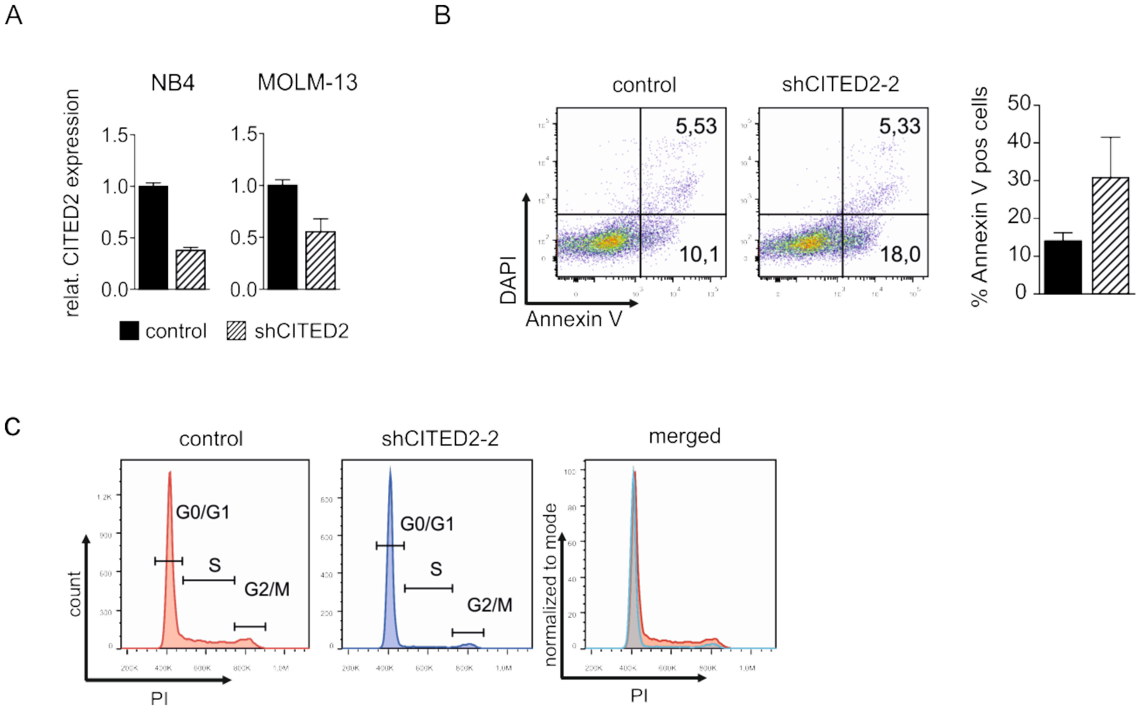

<u>Supplementary Figure S2 (Supplement to Figure 2)</u>

Loss of CITED2 triggers apoptosis in leukemic cells

(A) Q-PCR analysis for CITED2 in NB4 and MOLM-13 cells transduced with a lentiviral construct for downregulation of CITED2. Error bars indicated triplicates of representative Q-PCR. **(B)** Annexin V staining of MOLM-13 cells 4 days after transduction with shCITED2-2. FACS plots and bar graph indicate the percentage of Annexin V positive cells. Error bars indicate s.d.; n=2. (**C**) Cell cycle analysis of control- and shCITED2-2-transduced MOLM-13 cells.

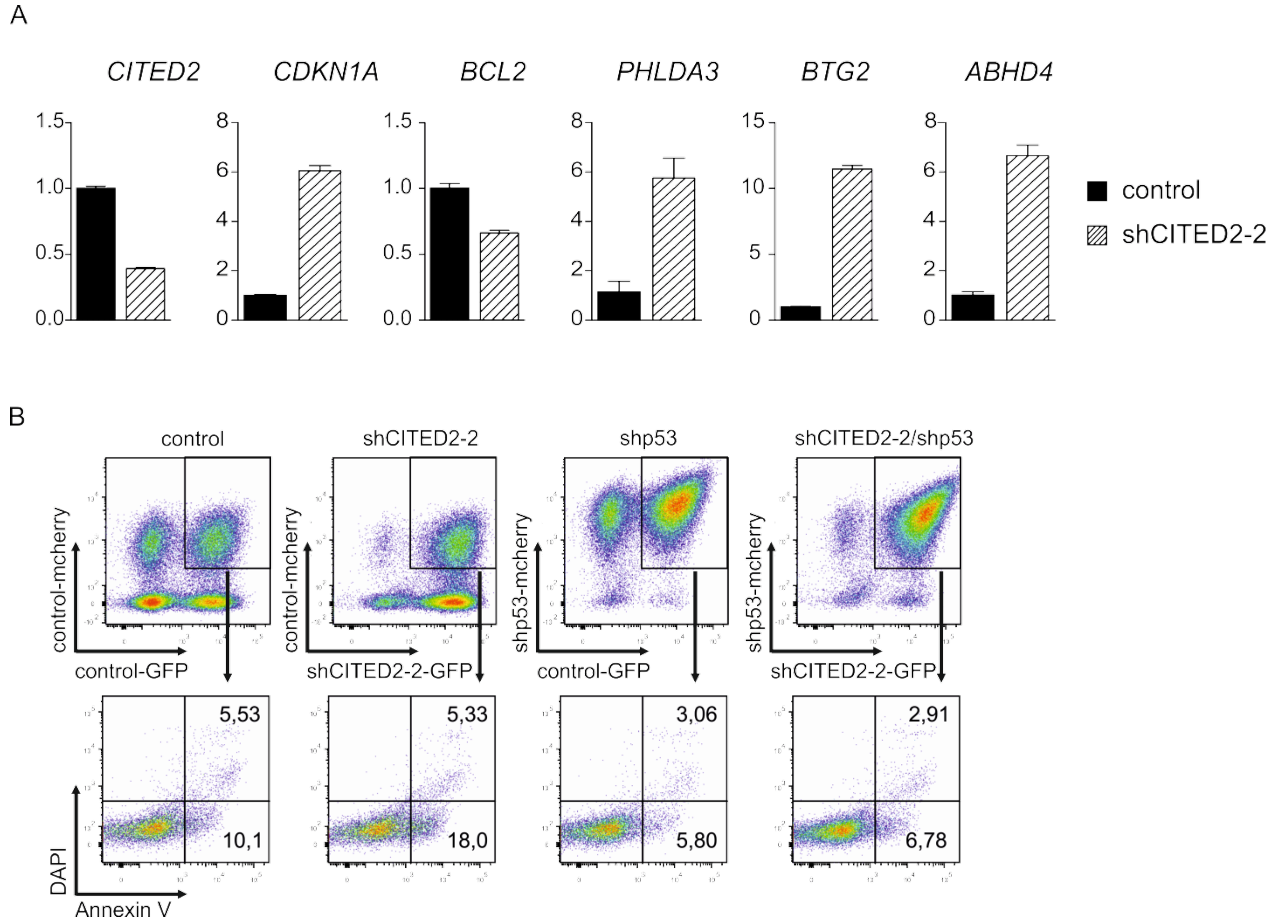

Supplementary Figure S3 (Supplement to Figure 3)

Cell death upon knockdown of CITED2 is triggered by a p53-dependent pathway

(A) Q-PCR of p53 targets in NB4 cells 4-5 days after transduction with the shCITED2-2 vector. Error bars indicated triplicates of representative Q-PCR. **(B)** MOLM-13 cells were double-transduced with GFP and mCherry expressing shRNA vectors to knockdown CITED2 and/or p53 and stained for Annexin V 4-5 days after transduction. FACS plots indicating the percentage of Annexin V positive cells are shown.

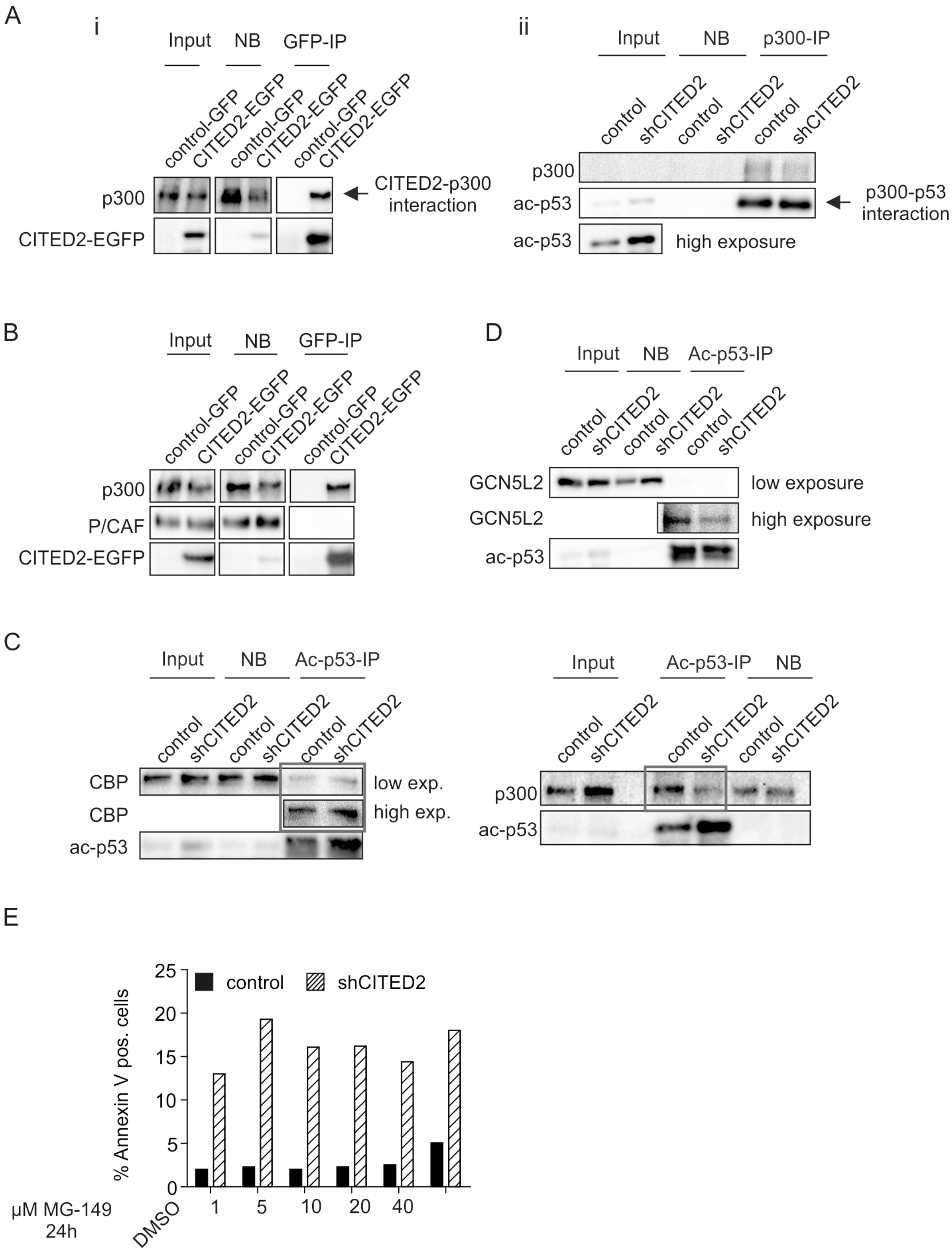

Supplementary Figure S4 (Supplement to Figure 4)

Increased p53 activation in CITED2 knockdown cells is not directly mediated by CBP/p300

(A)-(D) Western blot analysis of co-immunoprecipitation (IP) experiments of sorted GFP^+ NB4 cells with indicated antibodies **(Ai)** GFP co-IP of CITED2:EGFP-expressing cells. Black arrow indicates CITED2-

p300 interaction. **(Aii)** p300 co-IP of shCITED2-transduced cells. Black arrow indicates p300-p53 interaction. **(B)** GFP co-IP of CITED2:EGFP-expressing cells indicating that there is no interaction between CITED2 and P/CAF. **(C)** Ac-p53 co-IP of shCITED2-transduced cells. Red rectangle highlights the lanes indicating ac-p53-CBP interaction (left panel) or ac-p53-p300 interaction (right panel). **(D)** Ac-p53 co-IP of shCITED2-transduced NB4 cells demonstrating that there is no change in p53-GCN5L2 interaction upon CITED2 knockdown. **(E)** Percentage of Annexin V positive cells of control- or shCITED2-transduced MOLM13 cells after incubation with indicated concentration of the inhibitor MG-149 for 24h.

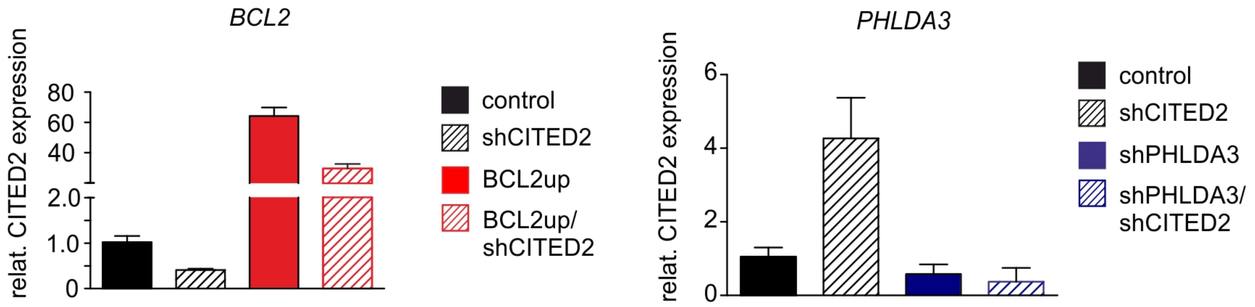

Supplementary Figure S5 (Supplement to Figure 5)

CITED2 knockdown mediates decrease in AKT-signaling in a p53/PHLDA3/BCL2 dependent manner

Q-PCR for BCL2 (left panel) and PHLDA3 (right panel) in MOLM-13 cells that were transduced with a short hairpin construct against *CITED2* in combination with a lentiviral construct for overexpression of BCL2 or a short hairpin against *PHLDA3.* Error bars indicated triplicates of representative Q-PCR.

Supplementary Table 1

Table lists primer sequences that have been used for Q-PCR .

name	forward primer 5'-3'	reverse primer 5'-3'
RPL27	TCCGGACGCAAAGCTGTCATCG	TCTTGCCCATGGCAGCTGTCAC
RPS11	AAGATGGCGGACATTCAGAC	AGCTTCTCCTTGCCAGTTTC
CITED2	CTTTGCACGCCAGGAAGGTC	CGCCGTAGTGTATGTGCTCG
CDKN1A	ACTAGGCGGTTGAATGAGAG	AGGAAGTAGCTGGCATGAAG
p53	GAGATGTTCCGAGAGCTGAATGAGGC	TCTTGAACATGAGTTTTTTATGGCGGGAGG
BCL2	GAGGCTGGGATGCCTTTGTG	GGGCCAAACTGAGCAGAGTC
PHLDA3	GGACCCTCGTGTCCTAAACC	CACAAGCCAGAGGGAACAAC
SOX4	GACCTGAACCCCAGCTCAAA	GATCATCTCGCTCACCTCGG
TXNIP	TTGCGGAGTGGCTAAAGTG	TCACCTGTTGGCTGGTCTTC
BTG2	TGGGCTTAGGGAACCATCTCT	TTCAGCCAAGGAATACATGCAA
ABHD4	GGAAGCCAGGATCCTCCAGTGTCT	CCAAGGGGGTGCGGTCGTTT
CBP	AGCTTGCAGAGGTCTTTGAG	TCCCATAGCAGCACAAAGTC
p300	CCTTCCCAGCCTCAAACTAC	CAGCCATCACAGACGAATCC
HPRT	AGTTCTGTGGCCATCTGCTTAG	CGCCCAAAGGGAACTGATAGTC

Supplementary Table 2

Table lists short hairpin sequences used for RNAi-mediated knockdown of the indicated target genes

Short hairpin name	Target gene	sequence
shCITED2	*CITED2*	AAATCCGGCATGTAGTGGTTG
shCITED2-2	*CITED2*	ATGAACTGGGAGTTGTTAAAC
shp300	*EP300*	TAATTCATCTGGTAAGTCG
shCBP	*CREBBP*	TCGCCACGTCCCTTAGTAACCA
shPHLDA3	*PHLDA3*	CAGCCTGGTACCAAAAGGAGTA
